# Machine learning applications on neonatal sepsis treatment: a scoping review

**DOI:** 10.1186/s12879-023-08409-3

**Published:** 2023-06-29

**Authors:** Colleen O’Sullivan, Daniel Hsiang-Te Tsai, Ian Chang-Yen Wu, Emanuela Boselli, Carmel Hughes, Deepak Padmanabhan, Yingfen Hsia

**Affiliations:** 1 School of Pharmacy, Queen’s University Belfast, Belfast, UK; 2 Centre for Neonatal and Paediatric Infection, St. George’s, University of London, London, UK; 3 School of Pharmacy, Institute of Clinical Pharmacy and Pharmaceutical Sciences, College of Medicine, National Cheng Kung University, Tainan, Taiwan; 4 Department of Pharmacy, National Cheng Kung University Hospital, College of Medicine, National Cheng Kung University, Tainan, Taiwan; 5 Department of Pediatrics, V. Buzzi Children’s Hospital, University of Milan, Milan, Italy; 6 School of Electronics, Electrical Engineering and Computer Science, Queen’s University Belfast, Belfast, UK

**Keywords:** Neonate, Bloodstream Infection, Machine Learning, Antibiotic, Antimicrobial Resistance

## Abstract

**Introduction:**

Neonatal sepsis is a major cause of health loss and mortality worldwide. Without proper treatment, neonatal sepsis can quickly develop into multisystem organ failure. However, the signs of neonatal sepsis are non-specific, and treatment is labour-intensive and expensive. Moreover, antimicrobial resistance is a significant threat globally, and it has been reported that over 70% of neonatal bloodstream infections are resistant to first-line antibiotic treatment. Machine learning is a potential tool to aid clinicians in diagnosing infections and in determining the most appropriate empiric antibiotic treatment, as has been demonstrated for adult populations. This review aimed to present the application of machine learning on neonatal sepsis treatment.

**Methods:**

PubMed, Embase, and Scopus were searched for studies published in English focusing on neonatal sepsis, antibiotics, and machine learning.

**Results:**

There were 18 studies included in this scoping review. Three studies focused on using machine learning in antibiotic treatment for bloodstream infections, one focused on predicting in-hospital mortality associated with neonatal sepsis, and the remaining studies focused on developing machine learning prediction models to diagnose possible sepsis cases. Gestational age, C-reactive protein levels, and white blood cell count were important predictors to diagnose neonatal sepsis. Age, weight, and days from hospital admission to blood sample taken were important to predict antibiotic-resistant infections. The best-performing machine learning models were random forest and neural networks.

**Conclusion:**

Despite the threat antimicrobial resistance poses, there was a lack of studies focusing on the use of machine learning for aiding empirical antibiotic treatment for neonatal sepsis.

**Supplementary Information:**

The online version contains supplementary material available at 10.1186/s12879-023-08409-3.

## Background

Sepsis is a significant cause of health loss worldwide. This is particularly true for neonates as over three million cases of neonatal sepsis were reported worldwide [1]. It has been estimated that sepsis and meningitis account for 6.8% of newborn deaths globally, making them important causes of neonatal morbidity and mortality [2]. Sepsis and meningitis can be defined as early-onset (EOS), or late-onset (LOS), with different causative pathogens and risk factors associated with each type. EOS, infections occurring in the first 72 h of life, is associated with *group B streptococcus, Listeria, Enterococcus*, and *Escherichia coli* whereas LOS, infections occurring after 72 h of life, are associated with Gram-positives (such as *Coagulase-negative staphyloocci*, *Staphylococcus epidermidis* and *Staphylococcus aureus*) and Gram-negatives (such as *E. coli, Klebsiellapneumoniae* and *Pseudomonas*) [3]. Low gestational birthweight, prematurity, low Apgar score, prolonged rupture of membranes (PROM) and chorioamnionitis are associated with a greater risk of both EOS and LOS; however the use of central venous catheters, previous antimicrobial exposure and poor hand hygiene increase the risk of hospital-acquired LOS, which is associated with higher rates of antimicrobial resistance [3–6].

Antimicrobial resistance remains a global public threat. It has been estimated that 70% of neonatal bloodstream infections are untreatable with ampicillin and gentamicin which are recommended as first-line treatment by the World Health Organization (WHO) [7–10]. In the absence of accurate diagnosis and treatment of sepsis, those who survive risk delayed growth and development [11]. The clinical presentations of neonatal sepsis can be difficult to identify and consequently challenging to treat [12]. The gold standard for a definitive diagnosis of neonatal sepsis is the isolation of pathogenic bacteria from a blood culture, which should be performed before giving the first dose of antibiotic [3]. However, sepsis can present with non-specific clinical signs and rapidly progress to multisystem organ failure without appropriate treatment [3]. For this reason, it is recommended to initially prescribe empiric antibiotic therapy for suspected neonatal sepsis. The empiric therapy consists of ampicillin plus an aminoglycoside, such as gentamicin, or cefotaxime [3]. Different antibiotics may be prescribed if antibiogram data show bacterial resistance patterns to the original prescription.

The current management of diagnosing and treating neonatal sepsis is expensive and time consuming. Furthermore, many resource-limited settings may not have the laboratory facilities or are understaffed in microbiology departments [13]. The absence of culture susceptibility testing increases the likelihood of receiving an inappropriate or ineffective course of antibiotic treatment. Antimicrobial resistance will invariably be exacerbated by overprescribing and the incorrect use of empirical antibiotics [14–16]. High levels of resistance for ampicillin and gentamicin have been reported in Gram-negative pathogens such as *K. pneumoniae* and *E. coli* in low-middle income countries (LMICs) [7–9, 17]. The high resistance rate for these antibiotics may lead to the use of carbapenems and third-generation cephalosporins as first-line treatment for neonatal sepsis. Despite the WHO guidance, a study has shown only 20% of neonates received WHO-recommended first-line treatment for neonatal sepsis in 41 countries and meropenem was predominately prescribed as first-line treatment in LMICs [9].

In the past decade, electronic health records (EHRs) have been extensively utilised for health care, including antibiotic stewardship research and to monitor antibiotic use [18]. In order to handle the complexity of EHRs, machine learning (ML) techniques have gained popularity as a powerful tool to identify data patterns and perform accurate predictions. ML is an analytical tool devised from disciplines like computer science, statistics, and mathematics for spotting patterns in data and exploiting these patterns to address a task [19]. ML aims to develop a model that best describes the available dataset, and the resulting model will improve automatically through the experience of encountering increasing amounts of data [19]. Studies have demonstrated the clinical value of applying ML for image processing to identify early signs of diseases and cancer diagnosis [20, 21].

Numerous studies have used EHRs to create prediction models to improve diagnosis, develop new medicine, and improve care [22–24]. There is a plethora of information to be mined due to the increase volume of data. For data scientists and healthcare professionals desiring to harness the vast and complex healthcare data, the development of machine learning technology is especially useful [25]. ML has been demonstrated to predict the likelihood of acquiring *Clostridium difficile* infections in hospitals and to predict patients with a significant risk of developing septic shock in adult populations [26, 27]. ML technology has already been used to aid clinicians in determining the most appropriate empiric antibiotic to prescribe for different infectious diseases in adult populations [28–30].

Classification ML models such as naïve Bayes classifier, k-nearest neighbours, support vector machines, decision tree methods (e.g. boosted decision trees and random forest), and the deep learning technique neural networks can handle categorial data well and may provide greater opportunities to address diagnosing and treating neonatal sepsis [19, 31–34]. The aim of this scoping review was to summarise the application of ML on neonatal sepsis treatment including the parameters that are required to perform ML, the common ML techniques applied, and to reflect on future directions.

## Methods

Three databases (PubMed, Embase and Scopus) were searched to identify relevant literature published from inception to 26^th^ November 2022. The Medical Subject Headings (MeSH) terms and keywords related to ML, neonatal sepsis, and antibiotics were searched. The search strategies were carried out with the following concepts: “sepsis”, “neonates”, “antibiotics”, and “machine learning” with slight adjustments made as suitable to each database (Appendices 1–3). Articles applying ML techniques to guide diagnosis, antimicrobial resistance, or treatment of neonatal sepsis were included. We included studies which applied ML modelling to predict bloodstream infectious disease management (diagnosis and treatment) in neonates aged < 28 days. Studies published in languages other than English were excluded along with review articles, letter, conference proceeding, and animal studies.

Three reviewers (DHT. Tsai, ICY. Wu, and C. O’Sullivan) independently conducted the initial screening of titles/abstracts for inclusion. Full-text article screening and data extraction were performed independently. Any disagreements were resolved by consensus.

**Fig. 1:**
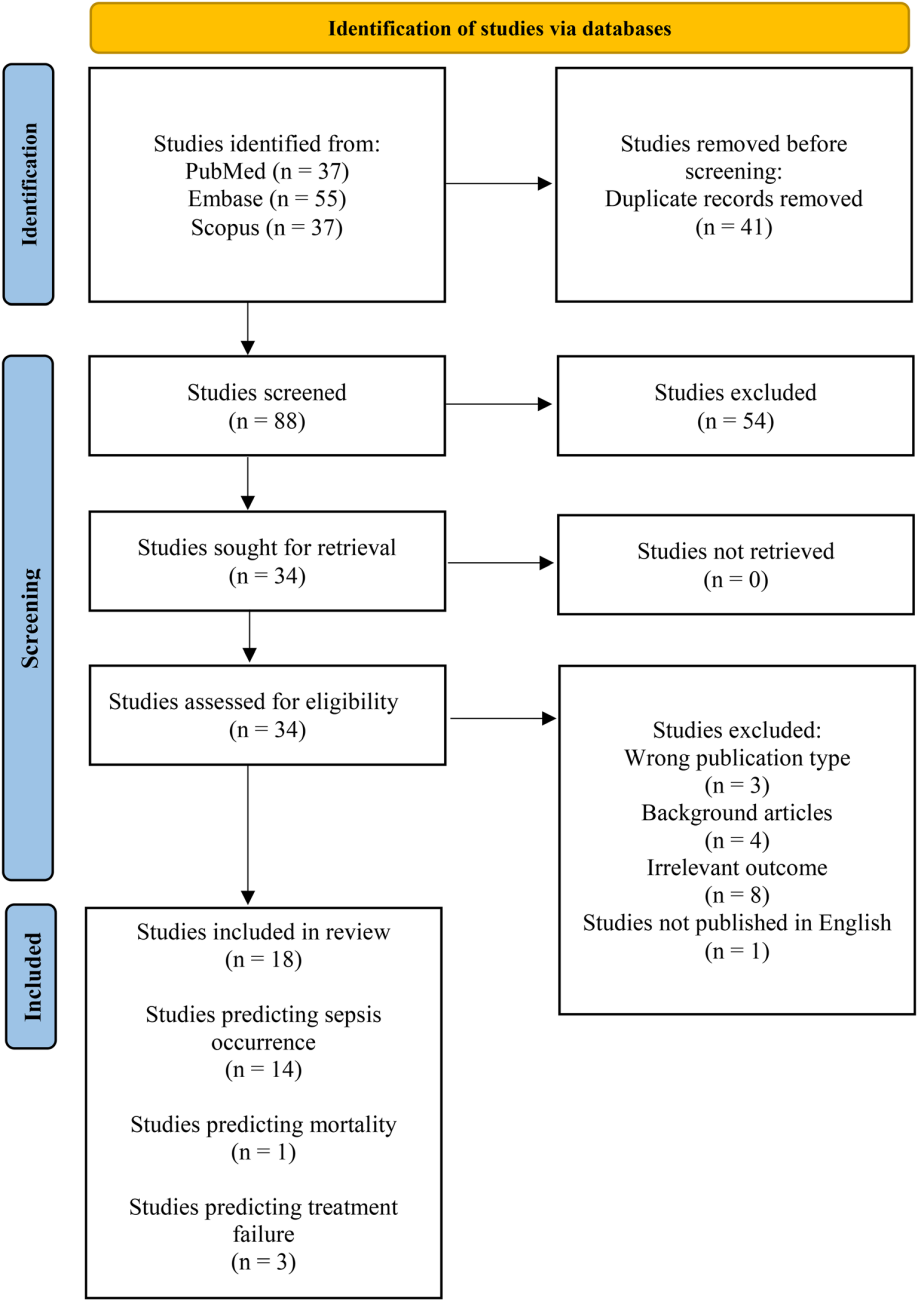
PRISMA flow diagram of numbers for studies identified in PubMed, Embase and Scopus

Details of each included study were extracted, including number of patients, country where the study was conducted, ML techniques, and missing data handling (e.g. imputation method). We did not perform a risk of bias assessment in our review due to a lack of appropriate bias assessment tools for ML studies. Figure 1 shows the flowchart of the articles included in the review.

## Results

From 88 records, 18 articles met the inclusion criteria and were included in the scoping review. A list of included studies is summarised in Table 1. Most studies were published within the past 5 years and were conducted in the United States of America (USA). The majority of studies developed models which predict the occurrence of neonatal sepsis in a timely manner while analysing the accuracy of certain biomarkers to predict neonatal sepsis [35–39]. One study focused on predicting mortality in hospitalised neonates with suspected sepsis [40]. Three studies focused on antibiotic treatment, of which one study focused on predicting treatment failure in antibiotic use [41]. Another developed a model to predict antimicrobial resistance in Enterobacterales isolates [42]. Oonsivilai et al. aimed to predict neonatal and paediatric bloodstream infections resistant to WHO-recommended antibiotics, a combination of ampicillin and gentamicin or ceftriaxone [43]. Generally, populations consisted of all infants admitted to the neonatal intensive care unit (NICU) with sepsis, although some studies focused on predicting EOS or LOS [37–39, 41]. Studies were mostly conducted using EHRs collected in a single centre, typically the NICU at a hospital located in the USA [37, 44–46]. One study collected data from 17 centres across multiple countries (The Netherlands, Canada, Czechia, and Switzerland) [39]. Thirteen studies used neonatal data from high income countries (HICs), and four studies used neonatal data from China and Cambodia. The scope of the review is limited as only studies published in English were included. It is possible that more studies report the use of machine learning in neonatal sepsis and were not included.

**Table 1:** Study characteristics of included studies of machine learning application on neonatal bloodstream infection

Study	Country	Settings^*^/Sample Size	Bloodstream infection definition	Missing data handling	Machine learning techniques^†^	Most important variables for predicting outcome	Outcome
Auguet et al. (2021)	Kenya, Cambodia, UK	NA / NA^‡^	NA	Complete case analysis	Bayesian generalised linear model	NA	Antimicrobial resistance in Enterobacterales isolates^‡^
Buhimschi et al. (2011)	USA	NBSCU / 180	Positive blood culture, laboratory data, symptoms of infection	NA	Bayesian latent-class analysis	NA	Early-onset sepsis
Cabrera-Quiros et al. (2021)	Netherlands	Medical centre / 358	Positive blood culture, symptoms of infection, intravenous antibiotics	NA	Logistic regressor, naïve Bayes, nearest mean classifier	RMSSD, AAR, RespSD RespIDR	Late-onset sepsis
Chakraborty et al.(2021)	UK	Multiple centres / 1,445	Positive blood culture, laboratory data, symptoms of infection	NA	NA	NA	Neonatal sepsis
Gojak et al. (2022)	Bosnia, Herzegovina	NA / 1000	NA	NA	Levenberg-Marquardt backpropagation algorithm, ANN	NA	Neonatal sepsis
Gordon et al. (2020)	USA	Children’s Hospital / 38	Positive blood culture, prolonged course of antibiotics	NA	Decision tree, random forest	Proline, N6-acetyllysine, taurine, cytidine, 2-ydroxyglutarate, ornithine, thymine, glutamate, α- ketoglutarate	Bacterial meningitis
Huang et al. (2021)	USA	Children’s Hospital / 2,357	Positive blood culture, antibiotics	NA	SVM, KNN, logistic regression, random forest, XGBoost	Neutrophil fluorescence intensity, neutrophil cell size, neutrophil dispersion width, gestational age, monocyte fluorescence intensity	Neonatal sepsis
Mani et al. (2014)	USA	Children’s Hospital / 299	Positive blood culture, symptoms of infection	Last observation carry forward (LOCF)	SVM, naïve Bayes classifier, KNN, decision tree, CART, random forest, logistic regression, lazy Bayesian rules	NA	Late-onset sepsis
Masino et al. (2019)	USA	NICU / 618	Positive blood culture, antibiotics	Mean value imputation	Logistic regression, naïve Bayes, SVM with a radial basis function KNN, Gaussian process, random forest, AdaBoost, and gradient boosting	NA	Neonatal sepsis
Metsvaht et al. (2009)	Estonia	2 NICU / 283	Laboratory data, symptoms of infection	Surrogate splitting	Multiple logistic regression, CART	Birthweight, C-reactive protein, white blood count, gestational age, platelet count, vasoactive treatment, blood glucose	Treatment failure in early onset neonatal sepsis
Oonsivilai et al. (2018)	Cambodia	Children’s Hospital / NA^‡^	NA	Complete case analysis	Logistic regression, decision trees, random forest, boosted decision trees, linear SVM, polynomial SVMs, radial SVMs, KNN	Time from admission to blood culture, patient age, hospital versus community-acquired infection, age-adjusted weight score	Antimicrobial resistance
Ramgopal et al. (2020)	USA	26 ED / 1,470	Positive blood culture	Complete case analysis	Logistic regression, random forest, SVM, ANN	Urinalysis	Serious bacterial infection
Sokou et al. (2022)	Greece	NICU / 291	Positive blood culture, laboratory data, signs of infection	Complete case analysis	Multivariable model through 10-fold cross-validation LASSO logit regression	Considerable change in skin colour	Neonatal sepsis
Stocker et al. (2022)	Netherlands, Canada, Czechia, Switzerland	17 centres / 685	Positive blood culture, antibiotics	Complete case analysis	Random forest	C-reactive protein, white blood count	Early-onset sepsis
Tabaie et al. (2021)	USA	NA / 2,749	Positive blood culture	NA	Bidirectional LSTM	Temperature	Serious infection
Tabaie et al. (2021)	USA	NA / 2,749	Positive blood culture, new antibiotic course	NA	XGBoost and ElasticNet	Max - diastolic blood pressure	Serious infection
Zhang et al. (2021)	China	Children’s Hospital / 70	Positive blood culture, laboratory data, non-specific or focal signs and symptoms	NA	Linear, LASSO, XGBoost	Serum creatinine, cerebrospinal fluid pyuric acid	Neonatal sepsis with meningoencephalitis
Zhang et al. (2018)	China	NA / 63	NA	NA	Random forest	Rapoport–Luebering glycolytic shunt; phospholipase C signaling	Neonatal sepsis

^*^NBSCU: Newborn Special Care Unit; NICU: Neonatal Intensive Care Unit; ED: Emergency Department

^†^CART: Classification and Regression Tree; LASSO: L1-penalised Least Absolute Shrinkage and Selection Operator; ANN: Artificial Neural Networks; XGBoost: Extreme Gradient Boosting; SVM: Support Vector Machine; KNN: K-Nearest-Neighbours; LSTM: Long Short-Term Memory

^‡^Aggregated data included neonatal data from Cambodia

The size of the datasets used in the included studies ranged from 38 to 2,749 patients. Missing data are inevitable for various reasons in EHRs. However, not all included studies explicitly described how missing data were handled. Few studies performed complete case analysis and variables with high levels of missing data were removed. Many neonates were not included for analysis if their key variables (such as age, weight, temperature, white cell count etc.) or blood culture results were not captured [35, 42, 46, 47]. Sample imputation was performed for two studies, in which missing data were replaced with either mean values or last observation carried forward (LOCF) [44, 45].

Risk factors with a strong association with neonatal sepsis, such as low gestational birthweight, prematurity, low Apgar score, prolonged rupture of membranes (PROM) and chorioamnionitis were included parameters in most models included in this review. However, the strongest predictors across most studies focusing on neonatal sepsis were gestational age, C-reactive protein levels, white cell count, platelet count, heart rate, and respiration [35, 36, 38, 39, 41, 45, 46]. Change in skin colour (pink was considered normal but a neonate turning green/grey was considered a significant change) was the strongest predictor in the Neonatal Sepsis Diagnostic (NeoSeD) model in Greece for predicting neonates with suspected sepsis [35]. Machine learning has also proven to be useful in the early detection of neonatal sepsis with meningoencephalitis, a complication associated with severe sepsis. Serum creatine and pyruvic acid, two metabolites found in cerebrospinal fluid are important parameters for differentiating neonates with septic shock and neonates with meningoencephalitis [36].

Blood glucose levels was found to be important for predicting antibiotic treatment failure and along with age in days and weight, days from hospital admission to blood sample was important for predicting WHO recommended antibiotic-resistant neonatal and paediatric bloodstream infections [41, 43]. Hospital-acquired infections was also an important variable for predicting ceftriaxone resistance and remarkably, the household size demographic proved to be useful in predicting ampicillin and gentamicin resistance [43]. A strong association between overcrowded households and infectious diseases has been evidenced, with higher rates of infectious disease transmission, and in turn antimicrobial resistant infections, being reported in households with more than eight persons [48, 49].

Blood pressure and temperature were important for predicting serious infections and urinalysis was important for predicting young febrile infants at risk of serious bacterial infections (including bloodstream) [47, 50, 51]. For predicting in-hospital neonatal mortality due to sepsis, the requirement of ventilator support at the onset of clinical suspected sepsis, feeding conditions and intravascular volume expansion were the strongest predictors [40].

Cross-validation was the most employed method to validate a ML model [35, 38–41, 43–46]. All studies reported the area under the receiver operating characteristics curve (AUC), and/or sensitivity and specificity as a metric to evaluate performance. The AUC for the best performing models for predicting sepsis in neonates ranged from 71.0–91.8% and the best performing models were logistic regression, boosted decision trees and random forest [35, 45, 46]. Sensitivity ranged from 38.0% to 94.0% and specificity ranged from 20.0% to 88.0% [38, 44–46]. Notably, not all ML models which gave high AUC values also provided high values for sensitivity and specificity; therefore it is important to include all these metrics to assess the full performance of the model [46]. Both logistic regression and decision trees performed well in predicting antibiotic treatment failure in neonates with sepsis, and random forest was the best performing model for predicting WHO recommended antibiotic resistant infections in neonates and children [41, 43]. Similarly, random forest had the highest specificity (74.9%) and highest sensitivity (98.6%) when predicting serious bacterial infections in febrile infants [47].

**Fig. 2:**
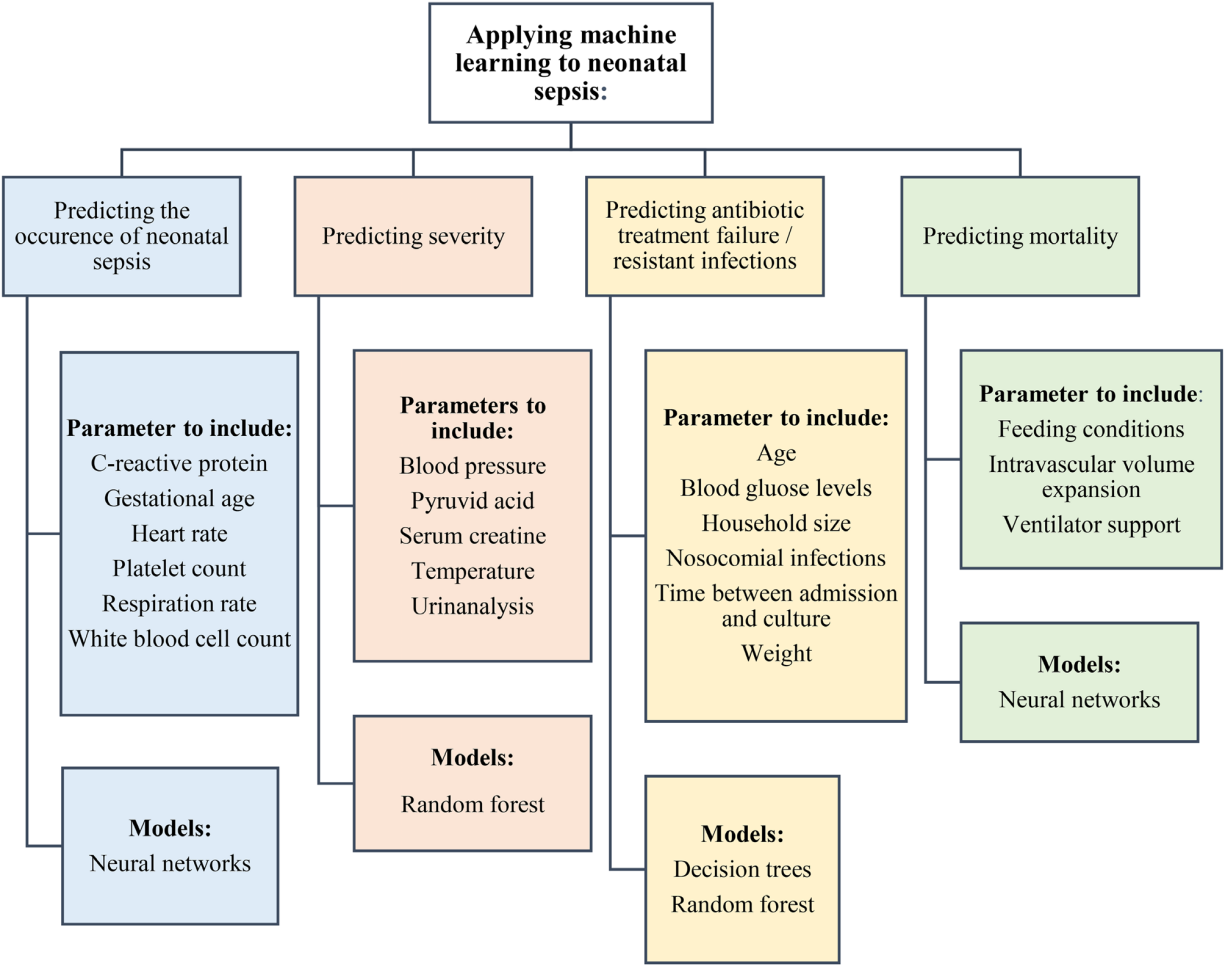
Schematic diagram of different applications of machine learning for neonatal sepsis including important parameters to include and which models are worth investigating for each task

The highest performing model in predicting neonatal sepsis mortality was a neural networks model (AUC: 92.3%) [40]. This model was highly accurate (95.6%) and specific (96.8%). However, this model differs from the models mentioned earlier, as a neural network model learns data in a similar sequence to the human brain [19]. In Bosnia and Herzegovina, neural networks were used to diagnose neonatal sepsis and performed much better than the machine learning models mentioned earlier, the model was highly sensitive and specific (98.8% and 95.5%) using data regularly collected at hospitals (body temperature, C-reactive protein, white blood cell count and platelet count) [52]. Neural networks generally provide more accurate models, but this accuracy is at the expense of transparency as these models are typically more complex [19]. Figure 2 shows the parameters to include for applying machine learning for neonatal sepsis identification and treatment, and which models have been reported to perform best for each task.

## Discussion

We performed this scoping review to summarise the published literature on ML application for neonatal sepsis diagnosis and treatment. From the included studies, the highest performing ML models in terms of AUC, sensitivity and specificity were the following: decision trees, random forest, and neural networks (a deep ML technique). Many included studies have failed to adequately handle missing data and did not explicitly describe if and how overfitting was prevented.

Typically, the performance of ML models improves when there are greater amounts of data. In Israel, a study by Yelin et al. developed ML models which could predict antimicrobial resistance for adults with urinary tract infections (UTIs) with great accuracy by linking 10-year longitudinal data of over 700,000 community-acquired UTIs with over 5,000,000 records of antibiotic treatment [29]. In comparison, the datasets of the studies included in this review were much smaller. This was expected due to the difficulties in acquiring neonatal data, therefore smaller datasets may limit the performance of ML models [12]. To ensure a ML model is providing sound and reliable results, it is important to validate the model. The recommended approach for developing ML models is to split the dataset into three sets: a training set, validation set, and testing set with cross-validation being the most widely employed validation ML technique for neonatal bloodstream infections [35, 38–41, 43–46].

The best data to train a ML model for neonatal bloodstream infections should include clinical data and laboratory data. All studies included in this review utilised demographic, clinical and laboratory data. The important clinical data to include were weight, age, days from admission to blood cultures taken, and information related to respiration such as ventilation status. Depending on the purpose of a ML model, the relevant laboratory data to include may differ. When developing ML models to predict the occurrence of a bloodstream infection in a neonate, biomarkers such as C-reactive protein and white blood cell count were essential. Antibiogram data should be included when developing ML models predicting resistant bloodstream infections [43, 53].

Using EHRs from a hospital to build a ML model is an approach that has been widely applied as observed in this review. However, the results from one single hospital cannot be generalised, meaning that the developed ML model is unlikely to give accurate predictions when the same ML model is applied to other hospitals, ultimately limiting the external validity [25]. Despite the significant threat antimicrobial resistance imposes globally, only three studies were identified which applied ML models to assist antibiotic treatment in this review. ML approaches could be employed to predict resistant patterns in neonatal bloodstream infections and in turn could assist clinicians in prescribing appropriate empiric antibiotic use.

In this scoping review, we limited our literature searches to three databases. We may miss some papers not indexed in these three databases. Also, we only included papers published in the English language. Currently, there are two reporting guidelines which were developed to facilitate the risk of bias assessment for prediction models: The Prediction Model Risk of Bias Assessment Tool Artificial Intelligence (PROBAST-AI) and the Transparent Reporting of a Multivariable Prediction Model of Individual Prognosis or Diagnosis (TRIPOD) statement [54–56]. As both suggested guidelines to evaluate the quality of prediction models are under development, we did not perform the risk of bias assessment in this scoping review.

### Future research

In the past few years, ML techniques have been used extensively to model EHRs and improve health care. Our review has shown that ML models have shown promise in assisting neonatal bloodstream infection management, of which the random forest model was the best model to predict the treatment outcome. To accurately identify patterns and produce predictions, it is essential to have a large volume of data. However, data collecting is generally labour-intensive, and it is even more challenging to collect data for neonates. This could be due to ethical complications or a lack of suitably trained staff, subsequently it results in smaller datasets being available [12]. Future studies should focus on developing and training models on bigger datasets and potentially validating these ML models via clinical trials similar to other computer based clinical tools [57].

A multimodal dataset is one approach to overcoming insufficient data. Linking data from multiple sources such as clinical and laboratory data, antibiogram data and genome sequencing data to create a multimodal dataset could improve disease detection, prognosis, and treatment for infectious diseases [12, 58]. However, genome sequencing is costly and may not be accessible to resource-limited regions. Another suggested approach would be to design a ML model which can predict laboratory results such as antibiogram results, eliminating labour-intensive laboratory data collection.

## Conclusion

This scoping review demonstrates the application of ML for neonatal bloodstream infection treatment. Despite the difficulties and barriers to obtaining data in this population, ML techniques have shown potential in making accurate diagnosis and treatment. However, there remains a lack of studies focusing on applying ML techniques to guide antibiotic selection to treat neonates with infectious diseases. Utilising ML techniques to assist clinicians in prescribing appropriate antibiotics for infectious diseases is still in its infancy. It warrants establishing a national and/or international network to build standardised structural data acquisition. This will bring ML application to a level where it can substantially reduce inappropriate antibiotic use in clinical practice.

## Electronic supplementary material

Below is the link to the electronic supplementary material.

Supplementary Material 1

Supplementary Material 2

## Data Availability

All data generated or analysed during this study are included in this published article [and its supplementary information files].

## List of Abbreviations

ANN: Artificial Neural Networks
AUC: Area Under the Receiver Operating Characteristics Curve
CART: Classification and Regression Tree
EHR: Electronic Healthcare Record
HIC: High Income Country
KNN: K-Nearest Neighbours
LASSO: L1-penalized Least Absolute Shrinkage and Selection Operator
LMIC: Low-Middle Income Country
LOCF: Last Observation Carried Forward
LSTM: Long Short-Term Memory
MeSH: Medical Subject Headings
ML: Machine Learning
NBSCU: Newborn Special Care Unit
NeoSeD: Neonatal Sepsis Diagnostic
NICU: Neonatal Intensive Care Unit
PROBAST-AI: The Prediction Model Risk of Bias Assessment Tool Artificial Intelligence
SVM: Support Vector Machine
TRIPOD: Transparent Reporting of a Multivariable Prediction Model of Individual Prognosis or Diagnosis
USA: United States of America
UTI: Urinary Tract Infection
WHO: World Health Organisation
XGBoost: Extreme Gradient Boosting

## References

[1] Fleischmann-Struzek C, Goldfarb DM, Schlattmann P, Schlapbach LJ, Reinhart K, Kissoon N (2018). The global burden of paediatric and neonatal sepsis: a systematic review. Lancet Respir Med.

[2] Liu L, Oza S, Hogan D, Chu Y, Perin J, Zhu J, Lawn JE, Cousens S, Mathers C, Black RE (2016). Global, regional, and national causes of under-5 mortality in 2000–2013;15: an updated systematic analysis with implications for the Sustainable Development Goals. Lancet.

[3] NICE. Neonatal infection: antibiotics for prevention and treatment. NG195. 2021. 10.1136/archdischild-2021-322349 34772670

[4] Araújo BC, Guimarães H (2020). Risk factors for neonatal sepsis: an overview. J Pediatr Neonatal Individualized Med (JPNIM).

[5] Soman M, Green B, Daling J (1985). Risk factors for early neonatal sepsis. Am J Epidemiol.

[6] Downey LC, Smith PB, Benjamin DK (2010). Risk factors and prevention of late-onset sepsis in premature infants. Early Hum Dev.

[7] Zaidi AKM, Huskins WC, Thaver D, Bhutta ZA, Abbas Z, Goldmann DA (2005). Hospital-acquired neonatal infections in developing countries. Lancet.

[8] Thaver D, Ali SA, Zaidi AKM (2009). Antimicrobial resistance among neonatal pathogens in developing countries. Pediatr Infect Dis J.

[9] Jackson C, Hsia Y, Basmaci R, Bielicki J, Heath PT, Versporten A, Goossens H, Sharland M (2019). Global divergence from World Health Organization treatment guidelines for neonatal and pediatric sepsis. Pediatr Infect Dis J.

[10] World Health Organization. Pocket Book of Hospital Care for Children: Second Edition. 2013. 24006557

[11] Schlapbach LJ, Aebischer M, Adams M, Natalucci G, Bonhoeffer J, Latzin P, Nelle M, Bucher HU, Latal B et al. Network tSN : Impact of sepsis on neurodevelopmental outcome in a Swiss national cohort of extremely premature infants. Pediatrics. 2011;128(2):348–57. 10.1542/peds.2010-3338 21768312

[12] Iregbu K, Dramowski A, Milton R, Nsutebu E, Howie SRC, Chakraborty M, Lavoie PM, Costelloe CE, Ghazal P (2022). Global health systems’ data science approach for precision diagnosis of sepsis in early life. Lancet Infect Dis.

[13] Bebell LM, Muiru AN (2014). Antibiotic use and emerging resistance: how can resource-limited countries turn the tide?. Glob Heart.

[14] Bronzwaer SL, Cars O, Buchholz U, Mölstad S, Goettsch W, Veldhuijzen IK, Kool JL, Sprenger MJ, Degener JE (2002). A European study on the relationship between antimicrobial use and antimicrobial resistance. Emerg Infect Dis.

[15] Costelloe C, Metcalfe C, Lovering A, Mant D, Hay AD (2010). Effect of antibiotic prescribing in primary care on antimicrobial resistance in individual patients: systematic review and meta-analysis. BMJ.

[16] Eliopoulos GM, Paterson DL, Rice LB (2003). Empirical antibiotic choice for the seriously ill patient: are minimization of selection of resistant organisms and maximization of individual outcome mutually exclusive?. Clin Infect Dis.

[17] Downie L, Armiento R, Subhi R, Kelly J, Clifford V, Duke T (2013). Community-acquired neonatal and infant sepsis in developing countries: efficacy of WHO’s currently recommended antibiotics - systematic review and meta-analysis. Arch Dis Child Fetal Neonatal Ed.

[18] Rezel-Potts E, Gulliford M. Electronic health records and antimicrobial stewardship research: a narrative review. Curr Epidemiol Rep. 2022;1–10. 10.1007/s40471-021-00278-1 PMC9303046 35891969

[19] James G, Witten D, Hastie T, Tibshirani R. An introduction to statistical learning. Volume 112. Springer; 2013.

[20] Chen JH, Asch SM (2017). Machine learning and prediction in medicine - beyond the peak of inflated expectations. N Engl J Med.

[21] Kourou K, Exarchos KP, Papaloukas C, Sakaloglou P, Exarchos T, Fotiadis DI (2021). Applied machine learning in cancer research: a systematic review for patient diagnosis, classification and prognosis. Comput Struct Biotechnol J.

[22] Melo MCR, Maasch JRMA, de la Fuente-Nunez C (2021). Accelerating antibiotic discovery through artificial intelligence. Commun Biol.

[23] Pesapane F, Codari M, Sardanelli F (2018). Artificial intelligence in medical imaging: threat or opportunity? Radiologists again at the forefront of innovation in medicine. Eur Radiol Exp.

[24] Lee CY, Chen YP (2019). Machine learning on adverse drug reactions for pharmacovigilance. Drug Discov Today.

[25] Wiens J, Shenoy ES (2018). Machine learning for healthcare: on the verge of a major shift in healthcare epidemiology. Clin Infect Dis.

[26] Wiens J, Campbell WN, Franklin ES, Guttag JV, Horvitz E. Learning data-driven patient risk stratification models for Clostridium difficile. Open Forum Infect Dis. 2014;1(2). 10.1093/ofid/ofu045 PMC4281796 25734117

[27] Henry KE, Hager DN, Pronovost PJ, Saria S (2015). A targeted real-time early warning score (TREWScore) for septic shock. Sci Transl Med.

[28] Feretzakis G, Loupelis E, Sakagianni A, Kalles D, Martsoukou M, Lada M, Skarmoutsou N, Christopoulos C, Valakis K, Velentza A et al. Using machine learning techniques to aid empirical antibiotic therapy decisions in the intensive care unit of a general hospital in Greece. Antibiotics. 2020;9(2). 10.3390/antibiotics9020050 PMC7167935 32023854

[29] Yelin I, Snitser O, Novich G, Katz R, Tal O, Parizade M, Chodick G, Koren G, Shalev V, Kishony R (2019). Personal clinical history predicts antibiotic resistance of urinary tract infections. Nat Med.

[30] Martínez-Agüero S, Mora-Jiménez I, Lérida-García J, Álvarez-Rodríguez J, Soguero-Ruiz C. Machine learning techniques to identify antimicrobial resistance in the intensive care unit. Entropy. 2019;21(6). 10.3390/e21060603 PMC7515087 33267317

[31] Cortes C, Vapnik V (1995). Support-vector networks. Mach Learn.

[32] Zhang Z. Introduction to machine learning: k-nearest neighbors. Ann Transl Med 2016; 4(11). 10.21037/atm.2016.03.37 PMC4916348 27386492

[33] Breiman L (2001). Random forests. Mach Learn.

[34] Freund Y, Schapire RE. Experiments with a new boosting algorithm. In: ICML*. *1996; Citeseer; 1996: 148–56.

[35] Sokou R, Ioakeimidis G, Piovani D, Parastatidou S, Konstantinidi A, Tsantes AG, Lampridou M, Houhoula D, Iacovidou N, Kokoris S (2022). Development and validation of a sepsis diagnostic scoring model for neonates with suspected sepsis. Front Pead.

[36] Zhang P, Wang Z, Qiu H, Zhou W, Wang M, Cheng G (2021). Machine learning applied to serum and cerebrospinal fluid metabolomes revealed altered arginine metabolism in neonatal sepsis with meningoencephalitis. Comput Struct Biotechnol J.

[37] Buhimschi CS, Bhandari V, Dulay AT, Nayeri UA, Abdel-Razeq SS, Pettker CM, Thung S, Zhao G, Han YW, Bizzarro M (2011). Proteomics mapping of cord blood identifies haptoglobin “switch-on” pattern as biomarker of early-onset neonatal sepsis in preterm newborns. PLoS ONE.

[38] Cabrera-Quiros L, Kommers D, Wolvers MK, Oosterwijk L, Arents N, van der Sluijs-Bens J, Cottaar EJE, Andriessen P, van Pul C (2021). Prediction of late-onset sepsis in preterm infants using monitoring signals and machine learning. Crit Care Explor.

[39] Stocker M, Daunhawer I, van Herk W, el Helou S, Dutta S, Schuerman FABA, van den Tooren-de Groot RK, Wieringa JW, Janota J, van der Meer-Kappelle LH et al. Machine learning used to compare the diagnostic accuracy of risk factors, clinical signs and biomarkers and to develop a new prediction model for neonatal early-onset sepsis. Pediatr Infect Dis J. 2022;41(3). 10.1097/INF.0000000000003344 34508027

[40] Hsu J-F, Chang Y-F, Cheng H-J, Yang C, Lin C-Y, Chu S-M, Huang H-R, Chiang M-C, Wang H-C, Tsai M-H (2021). Machine learning approaches to predict in-hospital mortality among neonates with clinically suspected sepsis in the neonatal intensive care unit. J Personalized Med.

[41] Metsvaht T, Pisarev H, Ilmoja M-L, Parm Ü, Maipuu L, Merila M, Müürsepp P, Lutsar I (2009). Clinical parameters predicting failure of empirical antibacterial therapy in early onset neonatal sepsis, identified by classification and regression tree analysis. BMC Pediatr.

[42] Auguet OT, Niehus R, Gweon HS, Berkley JA, Waichungo J, Njim T, Edgeworth JD, Batra R, Chau K, Swann J et al. Population-level faecal metagenomic profiling as a tool to predict antimicrobial resistance in Enterobacterales isolates causing invasive infections: an exploratory study across Cambodia, Kenya, and the UK. EClinic Med. 2021;36:100910. 10.1016/j.eclinm.2021.100910 PMC8173267 34124634

[43] Oonsivilai M, Mo Y, Luangasanatip N, Lubell Y, Miliya T, Tan P, Loeuk L, Turner P, Cooper BS (2018). Using machine learning to guide targeted and locally-tailored empiric antibiotic prescribing in a children’s hospital in Cambodia. Wellcome Open Res.

[44] Mani S, Ozdas A, Aliferis C, Varol HA, Chen Q, Carnevale R, Chen Y, Romano-Keeler J, Nian H, Weitkamp JH (2014). Medical decision support using machine learning for early detection of late-onset neonatal sepsis. J Am Med Inform Assoc.

[45] Masino AJ, Harris MC, Forsyth D, Ostapenko S, Srinivasan L, Bonafide CP, Balamuth F, Schmatz M, Grundmeier RW (2019). Machine learning models for early sepsis recognition in the neonatal intensive care unit using readily available electronic health record data. PLoS ONE.

[46] Huang B, Wang R, Masino AJ, Obstfeld AE (2021). Aiding clinical assessment of neonatal sepsis using hematological analyzer data with machine learning techniques. Int J Lab Hematol.

[47] Ramgopal S, Horvat CM, Yanamala N, Alpern ER. Machine learning to predict serious bacterial infections in young febrile infants. Pediatrics 2020;146(3). 10.1542/peds.2019-4096 PMC7461239 32855349

[48] Fonseca Lima EJ, Mello MJ, Albuquerque MF, Lopes MI, Serra GH, Lima DE, Correia JB (2016). Risk factors for community-acquired pneumonia in children under five years of age in the post-pneumococcal conjugate vaccine era in Brazil: a case control study. BMC Pediatr.

[49] Alividza V, Mariano V, Ahmad R, Charani E, Rawson TM, Holmes AH, Castro-Sánchez E (2018). Investigating the impact of poverty on colonization and infection with drug-resistant organisms in humans: a systematic review. Infect Dis Poverty.

[50] Tabaie A, Orenstein EW, Nemati S, Basu RK, Clifford GD, Kamaleswaran R (2021). Deep learning model to predict serious infection among children with central venous lines. Front Pead.

[51] Tabaie A, Orenstein EW, Nemati S, Basu RK, Kandaswamy S, Clifford GD, Kamaleswaran R (2021). Predicting presumed serious infection among hospitalized children on central venous lines with machine learning. Comput Biol Med.

[52] Gojak D, Gvožđar K, Hećimović Z, Smajović A, Bečić E, Deumić A, Bećirović LS, Pokvić LG, Badnjević A (2022). The use of artificial intelligence in the diagnosis of neonatal sepsis. IFAC-PapersOnLine.

[53] Corbin CK, Medford RJ, Osei K, Chen JH. Personalized antibiograms: machine learning for precision selection of empiric antibiotics. AMIA Jt Summits Transl Sci Proc. 2020;108–115. PMC7233062 32477629

[54] Andaur Navarro CL, Damen JAA, Takada T, Nijman SWJ, Dhiman P, Ma J, Collins GS, Bajpai R, Riley RD, Moons KGM (2021). Risk of bias in studies on prediction models developed using supervised machine learning techniques: systematic review. BMJ.

[55] Collins GS, Dhiman P, Andaur Navarro CL, Ma J, Hooft L, Reitsma JB, Logullo P, Beam AL, Peng L, Van Calster B (2021). Protocol for development of a reporting guideline (TRIPOD-AI) and risk of bias tool (PROBAST-AI) for diagnostic and prognostic prediction model studies based on artificial intelligence. BMJ Open.

[56] Collins GS, Reitsma JB, Altman DG, Moons KGM (2015). Transparent reporting of a multivariable prediction model for individual prognosis or diagnosis (TRIPOD): the TRIPOD Statement. BMC Med.

[57] Catho G, Sauser J, Coray V, Da Silva S, Elzi L, Harbarth S, Kaiser L, Marti C, Meyer R, Pagnamenta F (2022). Impact of interactive computerised decision support for hospital antibiotic use (COMPASS): an open-label, cluster-randomised trial in three Swiss hospitals. Lancet Infect Dis.

[58] Folgori L, Di Carlo D, Comandatore F, Piazza A, Witney AA, Bresesti I, Hsia Y, Laing K, Monahan I, Bielicki J (2021). Antibiotic susceptibility, virulome, and clinical outcomes in European infants with bloodstream infections caused by enterobacterales. Antibiotics.

