# Supplementary material for: Machine learning applications on neonatal sepsis treatment: a scoping review
Source: BMC Infect Dis. 2023 Jun 29;23:441. doi: 10.1186/s12879-023-08409-3 (PMC10308703; doi:10.1186/s12879-023-08409-3)
Supplement: Supplementary file 1 — Supplementary Material 1 [file 12879_2023_8409_MOESM1_ESM.docx]

| Concepts | Articles |
| --- | --- |
| Concept 1 | 385,745 |
| Concept 2 | 1,516,491 |
| Concept 3 | 4,830,642 |
| Concept 4 | 405,034 |
| Overall | 55 |

**Appendix 1.** Search terms used on **Embase** (1973-2022, 26/11/2022).

| **Concept 1**:  sepsis | AND | **Concept 2**:  Neonates | AND | **Concept 3**:  Antibiotics | AND | **Concept 4**:  Machine learning |
| --- | --- | --- | --- | --- | --- | --- |
| “sepsis”/exp |  | 'newborn'/exp |  | 'antibiotic agent'/exp |  | 'machine learning'/exp |
| **OR** |  | **OR** |  | OR |  | **OR** |
| “abdominal sepsis” |  | 'child, newborn' |  | 'antibiotic' |  | 'learning machine' |
| **OR** |  | **OR** |  | OR |  | **OR** |
| “focal sepsis” |  | 'full term infant' |  | 'antibiotic agent' |  | 'learning machines' |
| **OR** |  | **OR** |  | OR |  | **OR** |
| “intraabdominal sepsis” |  | 'human neonate' |  | 'antibiotic combination' |  | 'machine learning' |
| **OR** |  | **OR** |  | OR |  | **OR** |
| “sepsis” |  | 'human newborn' |  | 'antibiotic drug' |  | 'deep learning'/exp |
| **OR** |  | **OR** |  | OR |  | **OR** |
| “sepsis syndrome” |  | 'infant, newborn' |  | 'antibiotic residue' |  | 'deep learning' |
| **OR** |  | **OR** |  | OR |  | **OR** |
| “septic disease” |  | 'neonate' |  | 'antibiotic spectrum' |  | 'hierarchical learning' |
| **OR** |  | **OR** |  | OR |  | **OR** |
| “bloodstream infection”/exp |  | 'neonatus' |  | 'antibiotics' |  | 'artificial intelligence'/exp |
| **OR** |  | **OR** |  | OR |  | **OR** |
| “blood infection” |  | 'newborn' |  | 'antibiotics and their derivatives' |  | 'artificial intelligence' |
| **OR** |  | **OR** |  | OR |  | **OR** |
| “blood infections” |  | 'newborn baby' |  | 'antibiotics, combined' |  | 'machine intelligence' |
| **OR** |  | **OR** |  | OR |  |  |
| “blood stream infection” |  | 'newborn child' |  | 'antibiotics, folate antagonists' |  |  |
| **OR** |  | **OR** |  | OR |  |  |
| “blood stream infections” |  | 'newborn infant' |  | 'antibiotics, miscellaneous' |  |  |
| **OR** |  | **OR** |  | OR |  |  |
| “blood-borne infections” |  | 'newly born baby' |  | 'antibiotics, nitrofuran' |  |  |
| **OR** |  | **OR** |  | OR |  |  |
| “bloodstream infection” |  | 'newly born child' |  | 'antibiotics, oxalodinones' |  |  |
| **OR** |  | **OR** |  | OR |  |  |
| “bloodstream infections” |  | 'newly born infant' |  | 'combined antibiotic' |  |  |
|  |  | **OR** |  | OR |  |  |
|  |  | 'infant'/exp |  | 'antiinfective agent'/exp |  |  |
|  |  | **OR** |  | OR |  |  |
|  |  | 'infant' |  | 'anti bacterial agent' |  |  |
|  |  | **OR** |  | OR |  |  |
|  |  | 'baby'/exp |  | 'anti bacterial agents' |  |  |
|  |  | **OR** |  | OR |  |  |
|  |  | 'baby' |  | 'anti infective agents' |  |  |
|  |  | **OR** |  | OR |  |  |
|  |  | newborns |  | 'anti-bacterial agents' |  |  |
|  |  | **OR** |  | OR |  |  |
|  |  | 'infants'/exp |  | 'anti-infective agents' |  |  |
|  |  |  |  | OR |  |  |
|  |  |  |  | 'antibacterial' |  |  |
|  |  |  |  | OR |  |  |
|  |  |  |  | 'antibacterial agent' |  |  |
|  |  |  |  | OR |  |  |
|  |  |  |  | 'antibacterial drug' |  |  |
|  |  |  |  | OR |  |  |
|  |  |  |  | 'antibacterial spectrum' |  |  |
|  |  |  |  | OR |  |  |
|  |  |  |  | 'antiinfective agent' |  |  |
|  |  |  |  | OR |  |  |
|  |  |  |  | 'antimicrobial' |  |  |
|  |  |  |  | OR |  |  |
|  |  |  |  | 'antimicrobial agent' |  |  |
|  |  |  |  | OR |  |  |
|  |  |  |  | 'antimicrobial compound' |  |  |
|  |  |  |  | OR |  |  |
|  |  |  |  | 'antimicrobial drug' |  |  |
|  |  |  |  | OR |  |  |
|  |  |  |  | 'antimicrobial factor' |  |  |
|  |  |  |  | OR |  |  |
|  |  |  |  | 'antiseptic' |  |  |
|  |  |  |  | OR |  |  |
|  |  |  |  | 'antiseptic agent' |  |  |
|  |  |  |  | OR |  |  |
|  |  |  |  | 'chemotherapeutic agent' |  |  |
|  |  |  |  | OR |  |  |
|  |  |  |  | 'chemotherapeutic drug' |  |  |
|  |  |  |  | OR |  |  |
|  |  |  |  | 'chemotherapeutica' |  |  |
|  |  |  |  | OR |  |  |
|  |  |  |  | 'microbiological agent' |  |  |
|  |  |  |  | OR |  |  |
|  |  |  |  | 'antiseptics'/exp |  |  |
|  |  |  |  | OR |  |  |
|  |  |  |  | 'antisepsis'/exp |  |  |
|  |  |  |  | OR |  |  |
|  |  |  |  | 'antisepsis' |  |  |
|  |  |  |  | OR |  |  |
|  |  |  |  | antibacterials |  |  |

|  |
| --- |

| Concepts | Articles |
| --- | --- |
| Concepts 1 | 5,562,261 |
| Concepts 2 | 1,614,110 |
| Concepts 3 | 2,201,826 |
| Concepts 4 | 234,037 |
| Overall | 37 |

**Appendix 2.** Search terms used on **PubMed** (1992-2022, 26/11/2022).

| **Concept 1**:  sepsis | AND | **Concept 2**:  Neonates | AND | **Concept 3**:  Antibiotics | AND | **Concept 4**:  Machine learning |
| --- | --- | --- | --- | --- | --- | --- |
| sepsis |  | newborn |  | “anti-Bacterial Agents“[mh] |  | “machine learning“[mh] |
| **OR** |  | **OR** |  | **OR** |  | **OR** |
| septic |  | infant |  | antibiotic |  | “artificial Intelligence”[mh] |
| **OR** |  | **OR** |  | **OR** |  | **OR** |
| bloodstream |  | neonate |  | antibiotics |  | “machine learning” |
| **OR** |  | **OR** |  | **OR** |  | **OR** |
| blood |  | neonatus |  | antibacterial |  | “deep learning” |
| **OR** |  | **OR** |  | **OR** |  | **OR** |
| blood-borne |  | “newly born” |  | antibacterials |  | “artificial Intelligence” |
| **OR** |  | **OR** |  | **OR** |  | **OR** |
| sepsis[mh] |  | baby |  | antiseptic |  | “learning machine” |
|  |  | **OR** |  | **OR** |  | **OR** |
|  |  | “Infant, Newborn”[mh] |  | antisepsis |  | “learning machines” |
|  |  | **OR** |  | **OR** |  | **OR** |
|  |  | Infant[mh] |  | antiseptics |  | “hierarchical learning” |
|  |  |  |  | **OR** |  | **OR** |
|  |  |  |  | antiinfective |  | “machine Intelligence” |
|  |  |  |  | **OR** |  |  |
|  |  |  |  | antiinfectives |  |  |
|  |  |  |  | **OR** |  |  |
|  |  |  |  | antimicrobial |  |  |
|  |  |  |  | **OR** |  |  |
|  |  |  |  | antimicrobials |  |  |
|  |  |  |  | **OR** |  |  |
|  |  |  |  | chemotherapeutic |  |  |
|  |  |  |  | **OR** |  |  |
|  |  |  |  | chemotherapeutics |  |  |

| Concepts | Articles |
| --- | --- |
| Concepts 1 | 5,564,429 |
| Appendix | 1,805,129 |
| Concepts 3 | 1,440,589 |
| Concepts 4 | 976,262 |
| Overall | 37 |

**Appendix 3.** Search terms used on **Scopus** (1966-2022, 14/11/2022).

| **concept 1**:  sepsis | and | **concept 2**:  neonates | and | **concept 3**:  Antibiotics | and | **concept 4**:  Machine learning |
| --- | --- | --- | --- | --- | --- | --- |
| TITLE-ABS-KEY(sepsis) |  | TITLE-ABS-KEY(newborn) |  | TITLE-ABS-KEY(antibiotic) |  | TITLE-ABS-KEY(“machine learning”) |
| **OR** |  | **OR** |  | **OR** |  | **OR** |
| TITLE-ABS-KEY (septic) |  | TITLE-ABS-KEY(infant) |  | TITLE-ABS-KEY(antibiotics) |  | TITLE-ABS-KEY(“deep learning”) |
| **OR** |  | **OR** |  | **OR** |  | **OR** |
| TITLE-ABS-KEY (bloodstream) |  | TITLE-ABS-KEY(neonate) |  | TITLE-ABS-KEY(antibacterial) |  | TITLE-ABS-KEY(“artificial Intelligence”) |
| **OR** |  | **OR** |  | **OR** |  | **OR** |
| TITLE-ABS-KEY (blood) |  | TITLE-ABS-KEY(neonates) |  | TITLE-ABS-KEY(antibacterials) |  | TITLE-ABS-KEY(“learning machine”) |
| **OR** |  | **OR** |  | **OR** |  | **OR** |
| TITLE-ABS-KEY (blood-borne) |  | TITLE-ABS-KEY(“newly born”) |  | TITLE-ABS-KEY(antiseptic) |  | TITLE-ABS-KEY(“learning machines”) |
|  |  | **OR** |  | **OR** |  | **OR** |
|  |  | TITLE-ABS-KEY(baby) |  | TITLE-ABS-KEY(antisepsis) |  | TITLE-ABS-KEY(“hierarchical learning”) |
|  |  |  |  | **OR** |  | **OR** |
|  |  |  |  | TITLE-ABS-KEY(antiseptics) |  | TITLE-ABS-KEY(“machine Intelligence”) |
|  |  |  |  | **OR** |  |  |
|  |  |  |  | TITLE-ABS-KEY(antiinfective) |  |  |
|  |  |  |  | **OR** |  |  |
|  |  |  |  | TITLE-ABS-KEY(antiinfectives) |  |  |
|  |  |  |  | **OR** |  |  |
|  |  |  |  | TITLE-ABS-KEY(antimicrobial) |  |  |
|  |  |  |  | **OR** |  |  |
|  |  |  |  | TITLE-ABS-KEY(antimicrobials) |  |  |
|  |  |  |  | **OR** |  |  |
|  |  |  |  | TITLE-ABS-KEY(chemotherapeutic) |  |  |
|  |  |  |  | **OR** |  |  |
|  |  |  |  | TITLE-ABS-KEY(chemotherapeutica) |  |  |
